# Watermelon wilt disease: causes, harms, and control measures

**DOI:** 10.3389/fmicb.2025.1601130

**Published:** 2025-05-07

**Authors:** Yaoyao Tong, Haosheng Du, Jie Xiao, Buchan Zhou, Xiaojun Zheng, Yangwu Deng, Xianqing Zheng, Ming Chen

**Affiliations:** ^1^Jiangxi Provincial Key Laboratory of Environmental Pollution Prevention and Control in Mining and Metallurgy, Jiangxi University of Science and Technology, Ganzhou, China; ^2^Institute of Eco-Environment and Plant Protection, Shanghai Academy of Agricultural Sciences, Shanghai, China

**Keywords:** *Fusarium oxysporum* f. sp. *niveum*, continuous cropping obstacles, soil microbiome, biofumigation, microbial antagonism

## Abstract

Watermelon (*Citrullus lanatus L.*), a globally significant economic crop generating billions of dollars annually, faces severe production limitations due to persistent Fusarium wilt caused by continuous cropping. The disease emerges following watermelon cultivation, driven by the invasion of *Fusarium oxysporum* f. sp. *niveum*, the accumulation of allelochemicals in the rhizosphere, changes in soil properties, and disruptions to the soil microbial community. These factors interact complexly, influencing plant health and soil conditions. This review examines the causes and impacts of watermelon Fusarium wilt. It explores various control strategies, including developing resistant cultivars, adjusting planting systems and agricultural practices, soil fumigation, microbial inoculants, targeted fertilization, and reductive soil disinfection. Additionally, Future wilt control may leverage nanomaterial delivery systems for precisely targeted, environmentally sustainable fungicide applications in watermelon production. This review aims to establish a scientific foundation for preventing and controlling watermelon Fusarium wilt.

## 1 Introduction

### 1.1 A brief introduction of watermelon

Watermelon (*Citrullus lanatus L.*) ranks among the world’s most consumed fresh fruits, with global production reaching approximately 100 million tons annually according to the Food and Agriculture Organization of the United Nations (FAO, 2023). It is an annual dicotyledonous herb and a monoecious plant belonging to the gourd family *Cucurbitaceae*. Watermelon originated in southern Africa and has undergone roughly 4,000 years of domestication and breeding (Guo et al., 2019). Watermelons have been selectively improved into modern cultivated varieties known for their sweetness (Paris, 2015). In addition to its high water content (~92%) and provides multiple bioavailable nutrients, including carbohydrates (mainly fructose), proteins, vitamins, and essential minerals (Seymen et al., 2021; Karin and Melanie, 2009). This unique nutritional composition, particularly its high water content and electrolyte balance, makes it an ideal rehydration source. Given the projected increase in global temperatures and heatwave frequency under climate change scenarios, watermelon’s dual role as both a hydrating food and nutrient source suggests its agricultural importance will grow substantially in coming decades.

### 1.2 The continuous cropping obstacles of watermelon

Continuous cropping obstacles (CCOs) refer to the decline in crop yield or quality when the same crop or its related species are repeatedly grown on the same land (Wang K. et al., 2024). CCOs may arise due to three main factors: (i) shifts in microbial communities (Ma et al., 2023); (ii) allelopathic effects (Chen et al., 2023); and (iii) changes in nutrient availability (Zhang et al., 2024). As the global population grows and arable land becomes increasingly limited, CCOs have become a significant challenge in intensive, large-scale agricultural and horticultural systems (Tan et al., 2021). Watermelon production is particularly vulnerable to CCOs. Under continuous monoculture, watermelon is highly susceptible to these obstacles, exhibiting symptoms such as high seedling mortality, stunted growth, reduced vigor, wilting, plant death, and declines in both yield and quality (Zhang et al., 2022b). As a result, CCOs pose a major threat to the sustainable production of watermelon.

### 1.3 Remediation of watermelon CCOs

Over the past decades, various strategies have been explored to sustain watermelon production and mitigate CCOs. Most watermelon CCOs are caused by soilborne fungi, including *Fusarium oxysporum* f. sp. *Niveum* (FON), *Sclerotium rolfsii*, and *Macrophomina phaseolina*, which contribute to wilt disease in watermelon. The decade-long soil persistence of FON, mediated predominantly by its chlamydospores, represents a major obstacle for watermelon Fusarium wilt control (Zhang et al., 2015; McKeen and Wensley, 1961). The pathogen spreads through spores, which germinate and develop into hyphae. These hyphae penetrate vascular tissues through wounds, producing microconidia that facilitate reproduction. As the plant dies and decomposes, FON is released back into the soil, perpetuating the disease cycle (Rahman et al., 2021; Zhang et al., 2015). Chlamydospores formed by FON under stress are key to its decade-long soil persistence, which drives the recurrence and spread of wilt disease (Akhter et al., 2016; Hudson et al., 2021). FON can also spread through soil transfer, plant transplantation, seed sowing, and contaminated runoff. Once an area becomes contaminated, the risk of disease recurrence remains high (Rahman et al., 2021). Additionally, the presence of wilt disease alters soil microbial communities. Following infection, fungal diversity in the soil decreases, allowing FON to dominate the microbial ecosystem and intensifying the severity of wilt and other soilborne diseases (Xu et al., 2020).

Multiple management strategies have been developed to control pathogen proliferation through distinct mechanistic pathways: grafting resistant rootstocks induces systemic resistance, optimized crop rotation disrupts pathogen life cycles, intercropping with antagonistic species modifies rhizosphere microbiomes, while precision fertilization protocols and targeted soil fumigation techniques collectively alter physicochemical parameters critical for fungal survival (Ding et al., 2021; Li et al., 2019; Ling et al., 2015; Liu et al., 2023a; Lv and Yan, 2024; Yang et al., 2016).

### 1.4 Research status of watermelon wilt disease

**Figure 1:**
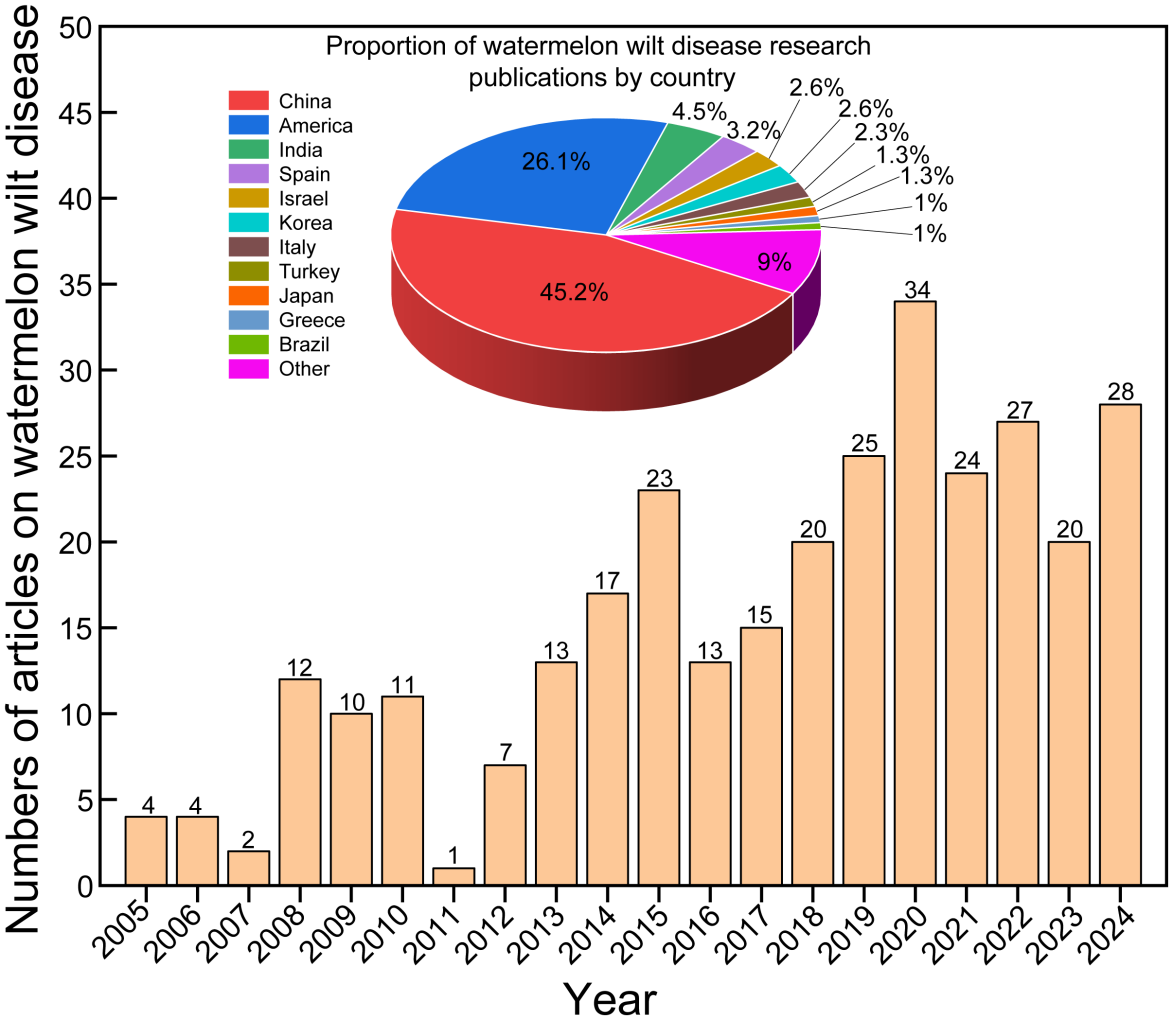
Number of publications related to watermelon and wilt disease from 2005 to 2024.

**Figure 2:**
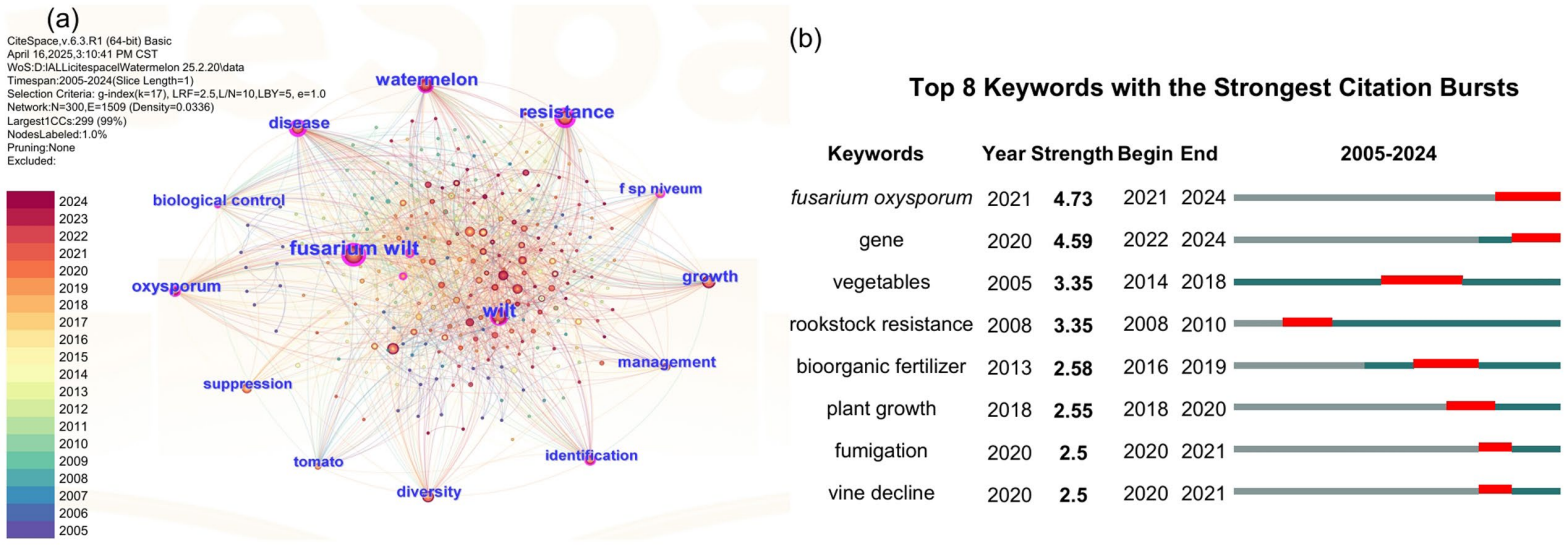
Co-occurrence network diagram **(A)** and the keyword bursts diagram **(B)** related to watermelon wilt disease.

To assess the research status of watermelon wilt disease, we conducted a literature search using the Web of Science platform. The search methodology is detailed in Supplementary Text S1. Using “wilt disease” as the search topic, we identified 10,590 publications from 2005 to 2024, among which only 2.93% (310/10,590) specifically addressed watermelon wilt disease, highlighting a critical research gap in this economically significant pathosystem (Figure 1). A co-occurrence network analysis of the keywords from these 310 articles showed that “watermelon Fusarium wilt” was the most frequently occurring keyword, indicating that watermelon Fusarium wilt is a central research focus in studies on wilt diseases affecting watermelon (Figure 2A). Further analysis of keyword bursts revealed that FON has been a primary subject in recent studies on watermelon wilt disease (Figure 2B).

Watermelon wilt caused by FON is the most severe soilborne disease affecting watermelon worldwide and can occur at all growth stages. However, the most critical period is from vine elongation to fruit setting, when the disease reaches its peak severity. The wilting of leaves (early stage) and vascular browning (late stage) result from the pathogen’s invasion process: root penetration, xylem colonization with toxin and enzyme production, and eventual vascular blockage causing permanent wilt (Zhang et al., 2005). Research on watermelon wilt disease continues to evolve. This review will focus on the tripartite determinants of Fusarium wilt pathogenesis: FON virulence, allelopathic interference, and abiotic stress potentiation. The review will also address the detrimental impacts of the disease, particularly plant damage coupled with yield and fruit quality reduction, and soil ecosystem disruption. Additionally, it will explore disease prevention and control strategies, including breeding resistant varieties, optimizing land management practices, and applying chemical or biological treatments.

## 2 Causes of watermelon wilt disease

The development of watermelon wilt disease is influenced by multiple factors resulting from interactions between plant roots, soil physicochemical properties, and microbial communities. These factors collectively contribute to disease onset and progression.

### 2.1 Direct pathogenesis: *Fusarium oxysporum f*. sp. *niveum*

Watermelon wilt disease is a soilborne disease primarily caused by pathogenic microorganisms inhabiting the rhizosphere. Upon infection, these pathogens produce phytotoxic compounds such as fusaric acid, lycomarasmin, and dehydrofusaric acid, which promote tissue colonization and block vascular conduits, thereby disrupting water transport and ultimately inducing plant wilting (Rahman et al., 2021; Michielse and Rep, 2009; Wang X. et al., 2024). Four distinct races of FON (race 0–3) have been identified based on their virulence and ability to infect different watermelon cultivars (Meru and McGregor, 2016). Race 0 is the least aggressive and only affects susceptible varieties lacking resistance genes, such as “Sugar Baby” and “Black Diamond.” Race 1 exhibits moderate pathogenicity and can infect some resistant cultivars, including “Charleston Gray.” Race 2 is highly virulent and can infect most watermelon varieties, causing severe wilt symptoms (Bruton et al., 2008). Race 3, first reported in 2006 and identified as a new race in 2009, represents the most aggressive strain of FON. As of 2018, no commercially available watermelon varieties have resisted race 3 (Amaradasa et al., 2018; Zhou et al., 2010).

Significant progress has been made in understanding the pathogenicity of FON. Recent studies indicate that various proteins, including protein kinase FonKin4, the pumilio protein family (PUF), and the ubiquitin–proteasome system (UPS), play essential roles in the ability of FON to infect and colonize host tissues (Gao et al., 2023; Noman et al., 2023b; Wang J. et al., 2024). Genes responsible for encoding these proteins have been identified as virulence factors. For example, *FonSMT3*, *FonAOS1*, *FonUBC9*, and *FonMMS21* are key components of the SUMOylation pathway, which regulates critical cellular and biochemical processes. Deleting these genes has been shown to significantly reduce the pathogenicity of FON (Azizullah et al., 2023; Azizullah et al., 2024).

Additionally, mutations in genes such as *FonNot2* have also been linked to decreased virulence (Dai et al., 2016). A complex network of multiple factors regulates the pathogenicity of FON. Further research is needed to elucidate the molecular mechanisms underlying FON infection in watermelon, which could aid in developing effective disease management strategies.

### 2.2 Effects of allelopathic chemicals

Allelopathic compounds, primarily secreted by plant roots and associated microorganisms, play a pivotal role in mediating interactions between plants and the rhizosphere microbiota (Supplementary Table S1). These chemicals, which include phenolic compounds, terpenes, flavonoids, polyacetylenes, and organic acids, can exert either stimulatory or inhibitory effects on neighboring organisms (Hao et al., 2010; Li et al., 2021; Polyak and Sukcharevich, 2019). In watermelon (*Citrullus lanatus L.*), allelopathic interactions have been increasingly recognized as a key factor influencing the onset and progression of Fusarium wilt, primarily caused by FON.

Watermelon roots predominantly exude phenolic acids as allelochemicals, including cinnamic acid, vanillic acid, coumaric acid, and ferulic acid (Wu et al., 2008a). These compounds have been demonstrated to act as autotoxins that inhibit seedling growth, with phytotoxic effects that may persist throughout the entire plant life cycle (Hao et al., 2007). Importantly, these autotoxins also influence the composition and stability of the rhizosphere microbial community. Disruption of microbial equilibrium can lead to reduced microbial antagonism against soilborne pathogens, thereby increasing the plant’s susceptibility to Fusarium wilt (Liu Q. et al., 2022).

Comparative studies across watermelon cultivars revealed significant differences in the types and relative proportions of phenolic acids. Wu et al. (2009) identified and classified 12 phenolic acids into antifungal and fungus-promoting groups, observing that the ratio of antifungal to fungus-promoting compounds was substantially lower in susceptible cultivars than in resistant ones. This finding suggests that a high abundance of fungus-promoting phenolic acids may compromise defense capacity in plants and contribute to disease development.

In addition to indirectly influencing FON through shifts in microbial dynamics, allelochemicals can also exert direct effects on the pathogen. Ferulic acid, at concentrations of 0.2 g·L^−1^, has been shown to stimulate hyphal growth, conidial germination, and mycotoxin production in FON, thereby exacerbating disease severity (Wu et al., 2010). Similarly, elevated levels (≥1.2 g·L^−1^) of specific amino acid have been found to promote FON growth and development (Liu et al., 2009). On the other hand, certain naturally occurring compounds may enhance plant resistance to Fusarium wilt. For instance, palmitic acid, a saturated fatty acid, has been shown to activate host defense mechanisms. Its application leads to increased reactive oxygen species (ROS) production and upregulation of defense-related enzymes, resulting in a significant reduction in FON abundance and enhanced early-stage resistance in watermelon seedlings (Kou et al., 2021; Ma et al., 2021; Li et al., 2022).

Taken together, these findings highlight the dual role of allelopathic compounds as both risk factors and potential modulators in Fusarium wilt pathogenesis. A deeper understanding of their biosynthesis, accumulation, and ecological impacts may offer novel insights for the development of sustainable disease management strategies.

### 2.3 Abiotic exacerbating factors

In addition to microbial pathogens and allelopathic compounds, abiotic environmental conditions significantly influence the onset and severity of watermelon wilt disease. These factors can either directly affect the physiological state of the host plant or indirectly alter the composition and functional stability of the rhizosphere microbiome, thereby modulating plant-pathogen interactions.

Soil physicochemical properties play a pivotal role in shaping disease dynamics. Soil pH, for instance, has been shown to affect both the growth of FON and the structure of microbial communities in the rhizosphere (hong et al., 2013; Lv et al., 2023). Suboptimal pH conditions, particularly acidification, can suppress beneficial microbial taxa while favoring pathogenic fungi (Das et al., 2022; Palmieri et al., 2023). Likewise, low levels of soil organic matter and poor aeration reduce the abundance and activity of antagonistic microorganisms that otherwise suppress FON proliferation (Zheng et al., 2024; Pommier, 2023). Soil compaction and poor drainage further exacerbate this imbalance by promoting anaerobic microenvironments, which impair root function and increase plant susceptibility to infection (Xu et al., 2025).

Nutrient availability and fertilization practices are also critical determinants of disease progression. Excessive nitrogen fertilization has been associated with increased disease incidence, likely due to enhanced pathogen growth and weakened host defenses (Sun et al., 2021). High nitrogen levels may stimulate the production of pathogen-derived phytotoxins such as fusaric acid and compromise systemic resistance in plants (Mur et al., 2017; Sun et al., 2020; Palmieri et al., 2023). In contrast, deficiencies in potassium and phosphorus, which are essential nutrients for root development and immune signaling, have been shown to compromise plant defense mechanisms and enhance susceptibility to soilborne pathogens (Dutta et al., 2024; Amtmann et al., 2008). Moreover, imbalanced nutrient regimes can modulate the production of root exudates, potentially altering the abundance and activity of pathogenic or beneficial microbes in the rhizosphere.

Environmental stressors such as extreme temperatures, drought, and flooding further intensify Fusarium wilt severity. Elevated soil temperatures not only enhance FON sporulation and virulence but also accelerate plant metabolic rates, often outpacing the activation of defense responses (Fawe et al., 1998; Venkatesh and Kang, 2019). Drought conditions disrupt root integrity and restrict the movement of defense-related phytohormones, while excessive soil moisture reduces oxygen availability, exacerbating root hypoxia and facilitating pathogen ingress (Jogawat et al., 2021; Gusain et al., 2024). These environmental fluctuations may also induce shifts in root exudate composition, indirectly promoting the accumulation of autotoxins or pathogen-favoring compounds in the rhizosphere (Zhalnina et al., 2018).

Collectively, these abiotic stressors interact in a complex and often synergistic manner with biotic factors, amplifying the risk of Fusarium wilt disease in watermelon. Integrated soil health management can suppress FON activity and reduce disease severity. Key strategies include adjusting pH, optimizing nutrients, improving drainage, and implementing crop rotations.

## 3 Harms of watermelon wilt disease

### 3.1 Plant damage and decline in yield and fruit quality

Once Fusarium wilt occurs in watermelon, it severely disrupts plant physiological functions, leading to significant reductions in both yield and fruit quality. Studies have shown that the pathogen FON exerts its pathogenicity primarily through the secretion of the secondary metabolite fusaric acid (FA). FA compromises the integrity of host cell membranes, induces excessive accumulation of mitochondrial reactive oxygen species (ROS), disrupts energy metabolism, and ultimately triggers programmed cell death (Xie et al., 2019). Additionally, FA significantly inhibits the biosynthesis of photosynthetic pigments in leaves, thereby reducing photosynthetic efficiency and causing leaf wilting and necrosis (Singh et al., 2017). As the initial site of infection, the root system also experiences substantial damage; FA suppresses root cell dehydrogenase activity, decreases membrane potential, and impairs water and nutrient uptake (Wu et al., 2008b). Moreover, FA-induced oxidative stress leads to lipid peroxidation, resulting in structural deterioration of leaf cells and further weakening the overall vitality of plants (Iqbal et al., 2023).

Such systemic damage directly compromises the yield formation process in watermelon. In continuous cropping systems, field incidence rates often reach 10–80% (Yang B. et al., 2024; Banerjee et al., 2025). Additionally, infected plants exhibit shortened growth periods, reduced fruit set, and impaired fruit development, which collectively intensify yield losses (Tong et al., 2025; Zheng et al., 2024; Pal et al., 2020). The soil persistence and accumulation ability of FON creates ongoing risks for watermelon production and complicates Fusarium wilt control.

### 3.2 Disruption of soil ecosystem

As the disease progresses, a cascade of physicochemical and biological alterations occurs within the rhizosphere. Notably, soil acidification becomes pronounced due to a gradual decline in pH, accompanied by elevated electrical conductivity and salinity, which together accelerate the process of soil salinization (Zheng et al., 2024). These changes not only compromise aggregate stability and reduce porosity but also degrade the overall structure of the soil, thereby impairing its ability to support healthy plant growth (Alekseeva et al., 2011; Pan et al., 2024). The resulting decrease in nutrient bioavailability often prompts growers to apply excessive amounts of chemical fertilizers in an attempt to sustain productivity. Overapplication of nitrogen-based fertilizers has been shown to suppress beneficial microbial taxa involved in key ecological functions, including antibiotic synthesis and nitrogen transformation (Fan et al., 2019; Zhang et al., 2022b). This imbalance disrupts microbial-mediated nutrient cycling and weakens soil disease resistance mechanisms. Moreover, excessive nitrogen inputs disturb the stoichiometric balance of carbon, nitrogen, and phosphorus in the rhizosphere, leading to nutrient imbalances that further constrain plant productivity (Cui et al., 2024).

Management practices involving the application of broad-spectrum fungicides, although aimed at disease suppression, often exert non-selective effects on soil microbiota. Such treatments can inhibit beneficial fungal populations and indirectly affect bacterial communities, particularly those involved in nitrogen cycling processes like ammonia oxidation (Carvalho, 2017; Luz et al., 2018; Meyer et al., 2024). Additionally, the potential for fungicide residues to accumulate in plant tissues and enter the food chain raises concerns about their broader ecological and human health implications (Lv et al., 2022). Collectively, these findings underscore the urgent need to adopt integrated soil and disease management strategies that prioritize ecological balance and long-term soil health.

## 4 Remediation measures of watermelon wilt disease

To minimize the yield and economic losses associated with watermelon production, the prevention and control of watermelon wilt disease have become a key research focus. Significant progress has been made in recent years. By managing interactions among plants, soil, and microorganisms, crop health and resistance to wilt disease can be significantly improved (Tong et al., 2024). Control strategies include breeding and cultivating highly resistant varieties, adjusting cropping systems, soil fumigation, applying microbial agents, optimizing fertilizer use, and reductive soil disinfestation.

### 4.1 Agroecological control measures

Agroecological strategies, including the development of resistant cultivars, Anti-stress grafting technique, and the optimization of cropping systems, offer sustainable solutions for managing Fusarium wilt in watermelon. The effectiveness of host resistance is closely related to both the watermelon genotype and the specific FON race (Supplementary Table S2). Several cultivars and germplasms such as “Charleston Gray,” “Longke No.13,” “PI 296341” and “PI 271769” have shown resistance to races 0, 1, or 2 (Wu et al., 2019; Bruton et al., 2008; Su et al., 2024; Wechter et al., 2012; Zhou and Everts, 2003), but highly resistant varieties remain limited, and no commercial cultivars are currently available for race 3 (Zhou et al., 2010; Song et al., 2017). Although conventional breeding methods and molecular techniques such as marker-assisted selection and QTL mapping have accelerated progress (Branham et al., 2019; Fall et al., 2018; Yang et al., 2009), developing cultivars with durable and broad-spectrum resistance remains challenging. By combining vigorous, stress-resistant rootstocks with high-yielding, high-quality scions, grafting not only enhances crop resistance to diseases but also improves tolerance to drought, salinity, and heavy metal stress. For instance, in watermelon grafting trials, using pumpkin as a rootstock significantly reduced the incidence of Fusarium wilt and increased the activity of antioxidant enzymes, thereby improving the plant’s adaptability to environmental stress (Mashilo et al., 2023).

Cropping system adjustments also play a key role in disease suppression. Crop rotation effectively suppresses Fusarium wilt through multiple mechanisms. Rice-watermelon rotation with intermittent flooding reduces FON populations (Ali et al., 2020). Wheat rotation significantly restructures soil microbial communities, enhancing beneficial taxa (e.g., increasing *Podospora* by 97%) while suppressing pathogens (e.g., reducing *Fusarium* by 89%), thereby improving soil health and disease suppression (Tian et al., 2023; Hu et al., 2025). Watermelon-garlic rotation increased yield by 67% and reduced wilt incidence by 50% (Yang et al., 2016), with garlic root exudates containing antimicrobial diallyl disulfide (DADS) identified as a key mechanism (Ren et al., 2018). Intercropping with wheat or aerobic rice can suppress pathogen growth through allelopathy and rhizosphere modification (Hao et al., 2010; Ren et al., 2008; Ren et al., 2016; Lv et al., 2018; Xu et al., 2015; Yu et al., 2019), though improper combinations may result in interspecies competition (Huang et al., 2017). Common intercropping combinations for watermelon include aerobic rice and wheat. Studies have shown that intercropping with aerobic rice suppresses pathogen spore production and modifies the microbial community in rhizosphere soil by influencing watermelon root exudates, thereby reducing disease severity (Hao et al., 2010; Ren et al., 2016). Other cultural practices, including delayed transplanting under higher temperatures (Keinath et al., 2019) and grafting onto resistant rootstocks (Ge et al., 2022; Ling et al., 2015; Toporek and Keinath, 2020), further contribute to disease mitigation. The integration of these approaches enhances system-level resilience and supports long-term, sustainable management of Fusarium wilt in watermelon.

### 4.2 Soil fumigation

Soil fumigation is a widely used method for controlling soilborne diseases (Sennett et al., 2022), including Fusarium wilt caused by FON. Fumigants evaporate and diffuse when applied to the soil, eliminating pathogens and beneficial microorganisms (Wang and Tang, 2024; Ge et al., 2021). Numerous studies have demonstrated the effectiveness of various fumigants in managing watermelon wilt disease. For example, ammonium bicarbonate (NH₄HCO₃) has been found to disrupt FON hyphae, causing fragmentation. Its effectiveness is further enhanced in acidic soils when combined with lime, increasing soil pH, improving nutrient availability, and boosting enzymatic activity (Li et al., 2019; Sun et al., 2015). Similarly, dazomet (C₅H₁₀N₂S₂) fumigation has increased soil phosphorus availability and enriched beneficial microorganisms, thereby enhancing watermelon resistance to FON infection (Zhu et al., 2020). Pic-Clor 60, a mixture of 1,3-dichloropropene (C₃H₄Cl₂) and chloropicrin (CCl₃NO₂), has been reported to reduce FON populations by over 90% while suppressing *Meloidogyne* spp. pathogens (Karki et al., 2022). However, the environmental persistence and non-target toxicity of synthetic fumigants necessitate alternative approaches (Tagele et al., 2021).

To address the limitations and environmental concerns associated with chemical fumigation, the development of safer and eco-friendly alternatives has gained increasing attention. Among these, biofumigation using cruciferous plants, particularly species from the *Brassica* genus, has shown promising potential due to their high glucosinolates content (Tagele et al., 2021). Although glucosinolates themselves have limited biocidal activity, their hydrolysis by myrosinase released upon tissue disruption produces isothiocyanates, which possess strong herbicidal and antimicrobial properties (Brennan et al., 2020; Zhang et al., 2019). Studies have shown that applying mustard plant extracts can reduce the incidence of Fusarium wilt in watermelon, enhance stress resistance, and promote seedling growth (Yuan et al., 2020). During biofumigation, the decomposition of *Brassica* tissues releases volatile compounds such as isothiocyanates and sulfur-containing substances, along with heat, which together inhibit pathogen growth (Liu et al., 2023b). Biofumigation combined with microbial inoculants improves soil nutrients, lowers pH, and reshapes fungal communities, mainly *Ascomycota* and *Basidiomycota* (95.14–96.17%), thereby creating a more favorable environment for watermelon growth (Chang et al., 2022). Taken together, these findings highlight the multifaceted benefits of biofumigation as a sustainable strategy for enhancing soil health and promoting watermelon productivity in environmentally responsible agricultural systems.

### 4.3 Application of microbial agents

**Figure 3:**
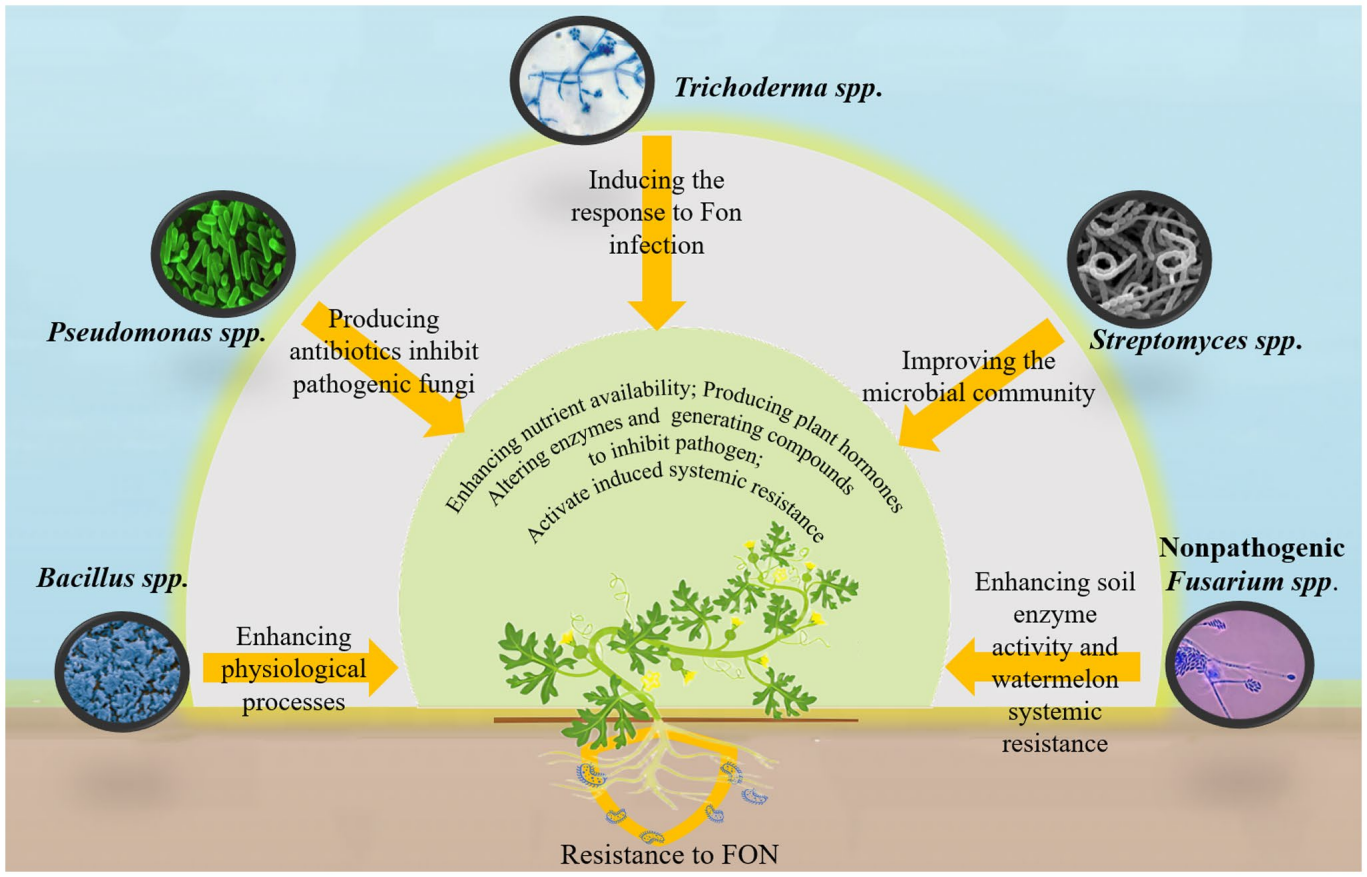
Biocontrol microorganisms help watermelon plants resist attacks by Fon.

The direct application of beneficial microbial agents to the soil is an effective strategy for mitigating watermelon wilt. Various microbial species, including *Bacillus* spp., *Pseudomonas* spp., *Trichoderma* spp., *Streptomyces* spp., and nonpathogenic *Fusarium* spp., have been utilized for pathogen control (Figure 3) (Faheem et al., 2015). These microorganisms promote plant health and growth through both direct and indirect mechanisms. Directly, they enhance plant development by improving nutrient availability, producing plant hormones, modifying enzyme activity, and generating antimicrobial compounds that inhibit or kill pathogens. Indirectly, certain microorganisms activate induced systemic resistance (ISR), strengthening plant immunity, making them more resistant to diseases and pests (Luo et al., 2023; Viaene et al., 2016).

Recent research has highlighted the potential of microbial-based technologies for controlling watermelon wilt. Noman et al. (2023a) developed biogenic manganese nanoparticles from *Bacillus megaterium* NOM14, which enhance various physiological processes in watermelon plants, thereby suppressing disease onset. Similarly, *Trichoderma asperellum* M45a has been shown to trigger defensive responses in watermelon roots against FON infection while increasing the diversity of beneficial rhizosphere bacteria (Zhang Y. et al., 2022). The solid fermentation products of *Streptomyces ahygroscopicus* strain 769 improve rhizosphere microbial community composition, creating a highly interconnected microbial network that enhances the expression of resistance-related and auxin-regulated genes in watermelon, ultimately reducing the incidence of FON infection (Ge et al., 2024). In addition, inactivated FON mycelium has significantly enhanced soil enzyme activity and systemic resistance in watermelon plants. This treatment also improves the abundance and diversity of beneficial rhizosphere microorganisms while substantially reducing FON populations and disease severity (Xie et al., 2021). However, microbial agents containing a single strain are not always effective. A combination of multiple strains may improve efficiency and stability. For example, a mixture of *Pseudomonas fluorescens* P4 and *Bacillus amyloliquefaciens* XY-13 has demonstrated antagonistic effects against 11 pathogenic fungi, including FON, with an inhibition rate of up to 78.17% against watermelon wilt (Yang D. et al., 2024). Similarly, a synthetic microbial consortium consisting of 16 core bacterial strains has been shown to mitigate watermelon wilt, with the synergistic interactions among these microorganisms playing a key role in disease suppression (Qiao et al., 2024).

### 4.4 Rational fertilization

Continuous cropping leads to the depletion and imbalance of soil nutrients, particularly in monoculture systems, which compromises plant resistance and increases susceptibility to soil-borne pathogens such as *Fusarium oxysporum* (Chen et al., 2018). Improper fertilization, especially the excessive and long-term application of chemical fertilizers, not only fails to restore soil health but also disrupts microbial communities. High concentrations of chemical inputs reduce microbial diversity, suppress beneficial taxa such as nitrogen-fixing and phosphate-solubilizing bacteria, and increase soil salinity and acidification (Bitew and Alemayehu, 2017). These changes create favorable conditions for the survival and proliferation of pathogenic fungi, thereby intensifying the risk of wilt disease (Fu et al., 2020; Zhang et al., 2022b).

In contrast, rational fertilization strategies, particularly those involving organic and bioorganic amendments, effectively alleviate soil nutrient stress and restore microbial balance. These inputs increase soil organic matter and improve the availability of key nutrients such as nitrogen, phosphorus, and potassium, which support plant development and metabolic resilience (Zhang et al., 2022a). More importantly, organic fertilization enhances the abundance and activity of beneficial microbial groups such as *Bacillus*, *Pseudomonas*, and *Trichoderma*, which are known for their biocontrol functions (Li et al., 2024). Additionally, organic fertilizers improve soil structure and buffer capacity, raising pH levels and reducing the accumulation of toxic ions (Liu et al., 2024). This creates a physicochemical environment less conducive to pathogen infection and colonization. The combined effects of microbial antagonism, improved nutrient dynamics, and enhanced root zone conditions contribute to a marked reduction in the incidence of watermelon wilt (Ling et al., 2014; Zhang et al., 2022b; Zhao et al., 2018). These findings underscore the mechanistic advantages of rational fertilization in promoting plant health and maintaining soil ecological stability.

### 4.5 Reductive soil disinfestation

**Figure 4:**
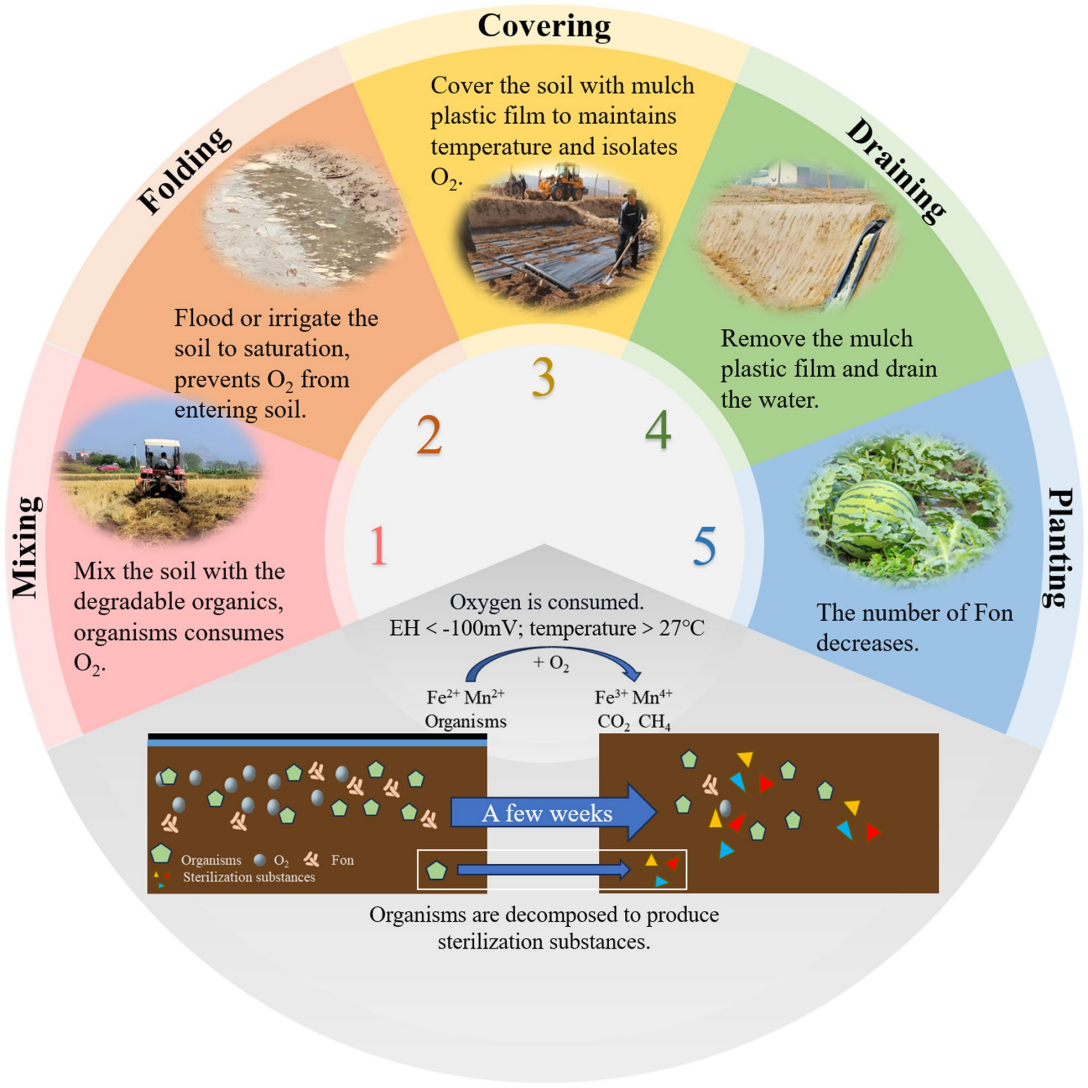
Schematic of the four-step reductive soil disinfestation (RSD) process.

Reductive soil disinfestation (RSD) is a soil treatment method that creates strong reducing conditions to eliminate aerobic pathogens. Also known as anaerobic soil disinfestation or biological soil disinfestation (Lopes et al., 2022), this technique was first developed by researchers in Japan (Shinmura, 2000) and the Netherlands (Blok et al., 2000). RSD typically consists of four key steps (Figure 4). First, easily degradable organic materials are evenly mixed into the soil. Second, the soil is flooded to saturation. Third, the soil is covered with a plastic mulch film for several weeks to maintain anaerobic conditions. Finally, the plastic film is removed, and excess water is drained to prepare the soil for planting (Ueki et al., 2018). The effectiveness of RSD is attributed to several mechanisms. Anaerobic and high-temperature conditions impose stress on pathogens. Anaerobic bacteria decompose organic matter, producing compounds harmful to pathogens (Momma et al., 2006). Additionally, the process leads to the release of iron and manganese ions, which further contribute to pathogen suppression (Momma et al., 2011). RSD also induces shifts in soil microbial communities, favoring the proliferation of beneficial microbes (Hewavitharana and Mazzola, 2016).

While these mechanisms collectively contribute to effective pathogen suppression, the transient nature of RSD-induced soil modifications poses challenges for long-term disease control, as observed in subsequent cropping cycles. Studies have shown that RSD significantly reduces bacterial diversity and alters microbial community composition. However, after watermelon replanting, bacterial diversity gradually recovers (Meng et al., 2019). Liu et al. (2018) showed that RSD treatment effectively suppresses FON growth while promoting beneficial soil microbiota. This not only prevents watermelon wilt but also enhances plant growth. However, after subsequent watermelon planting, the microbial community structure tends to revert to its original state, allowing FON populations to recover and regain pathogenicity. Researchers suggest that watermelon root exudates may play a role in driving this phenomenon. To extend the efficacy of RSD, some researchers have tested prolonged treatment durations at low temperatures (3.3°C–12.2°C), which have been shown to further improve soil microbial communities and physicochemical properties while suppressing soilborne pathogens (Liu L. et al., 2022). While RSD effectively controls watermelon wilt, the risk of disease recurrence in subsequent plantings remains. Further research is needed to better understand the mechanisms behind this process and to develop strategies for extending the duration of RSD treatment efficacy.

## 5 Conclusions and future perspectives

Watermelon wilt disease, caused by FON, represents a significant threat to global watermelon production, particularly in continuous cropping systems. The pathogenesis involves dynamic tripartite interactions among: (i) FON virulence factors, (ii) allelochemical-mediated rhizosphere feedbacks, and (iii) soil microbiome-physicochemical co-dysregulation, culminating in systemic host physiological disruption and yield depression.

Current management strategies, including resistant varieties, soil fumigation, microbial agents, and reductive soil disinfestation, have shown partial success but require optimization for improved efficacy and sustainability. Future research should focus on four key directions: First, advancing breeding programs through multi-omics approaches to develop durable resistant cultivars. Second, engineering precision microbiome solutions that simultaneously target pathogens, degrade allelochemicals, and enhance plant immunity. Third, exploring innovative nanotechnology applications for targeted delivery of antifungal compounds. Fourth, optimizing integrated soil health management practices tailored to regional conditions.

This comprehensive approach, balancing scientific innovation with practical application, will be crucial for ensuring the long-term sustainability of watermelon production while maintaining environmental integrity and meeting global food demands. Future studies should prioritize field validation of emerging technologies and economic feasibility assessments to facilitate widespread adoption.
