# Supplementary material for: Watermelon wilt disease: causes, harms, and control measures
Source: Front Microbiol. 2025 May 7;16:1601130. doi: 10.3389/fmicb.2025.1601130 (PMC12092397; doi:10.3389/fmicb.2025.1601130)
Supplement: Supplementary file 1 [file Data_Sheet_1.docx]

**Supporting Information**

**SI Text 1:** **The search methodology about publications in Web of Science platform**

Using "wilt disease" as the topic, the “Web of Science Core Collection” database was selected with "All" chosen under “Collections”, searching on the Web of Science platform. The initial results were filtered by publication years "2005-2024" and document type "Article", yielding 10,590 publications. Subsequent refinement by adding the keyword "watermelon" and selecting the 'include' option resulted in 310 publications. (Search conducted on February 21, 2025).

**Table S1 Common allelopathic substances in watermelon rhizosphere**

| Allelopathic substances | Structural formula | Effects | Reference |
| --- | --- | --- | --- |
| Salicylic acid | 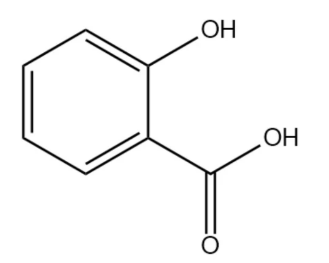 | An antifungal compound， a signal for systemic acquired resistance, can induce the expression of pathogenesis-related genes that encode proteins with antibacterial activity. | (Hao et al., 2010; Ren et al., 2016) |
| ρ-coumalic acid | 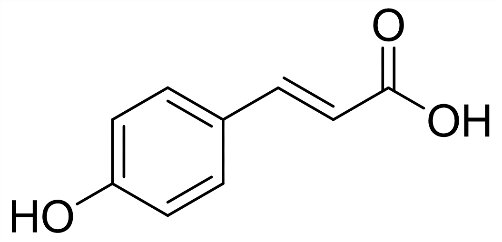 | An active substance with antibacterial properties that inhibits the growth of the germination and sporulation of Fon spores | (Ren et al., 2016; Wu et al., 2009) |
| Phthalic acid | 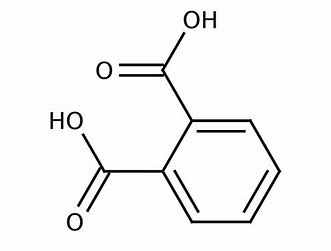 | Promoting the germination of watermelon seeds enhances the absorption and accumulation of nutrients and the activity of antioxidant enzymes in the roots in low concentrations. | (Hao et al., 2010; Li et al., 2022) |
| Palmitic acid | 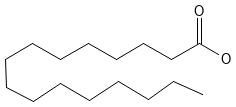 | An antifungal compound. Recruiting beneficial bacteria, changing the microbial composition, and inducing the systemic  resistance in watermelon against FON. | (Kou et al., 2021; Ma et al., 2021; Tong et al., 2024) |
| Cinnamic acid | 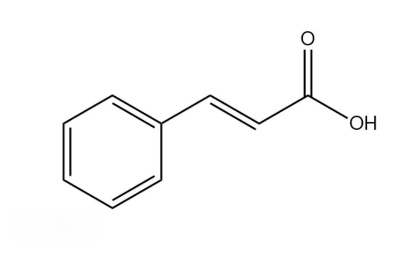 | An antifungal compound. Inhibitory effect on the mycelial growth of Fon, but promoting mycotoxin production and hydrolytic enzyme activity of Fon. | (Wu et al., 2008a) |
| Phenylalanine | 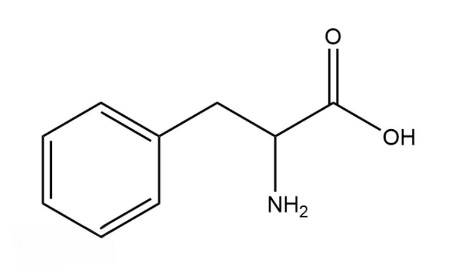 | A fungal-promoting compound. These are important nutrients for Fon and promote the growth of Fon spores at higher concentrations but inhibit them at lower concentrations. | (Liu et al., 2009; Tong et al., 2024) |
| Ferulic acid | 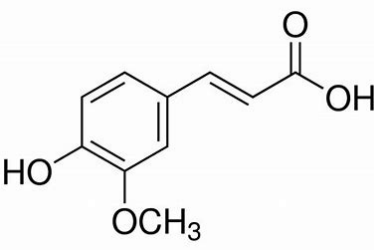 | A fungal-promoting compound. Stimulating the sporulation and germination of Fon spores, a precursor for phenylpropanoid derivatives, can exhibit antibacterial properties and enhance plant defense capabilities. | (Ren et al., 2016; Wu et al., 2010) |
| Alanine | 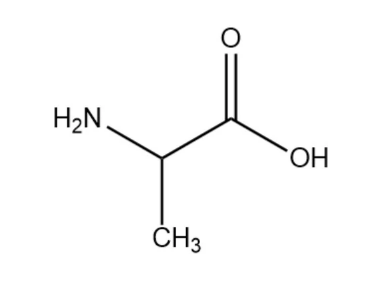 | A fungal promoting compound, with an obvious effect on the enhancement of spore germination and sporulation of FON | (Hao et al., 2010) |

**Table S2 Resistant varieties and germplasms of watermelon and characteristics**

| Varieties | Characteristics | Reference |
| --- | --- | --- |
| Charleston Gray  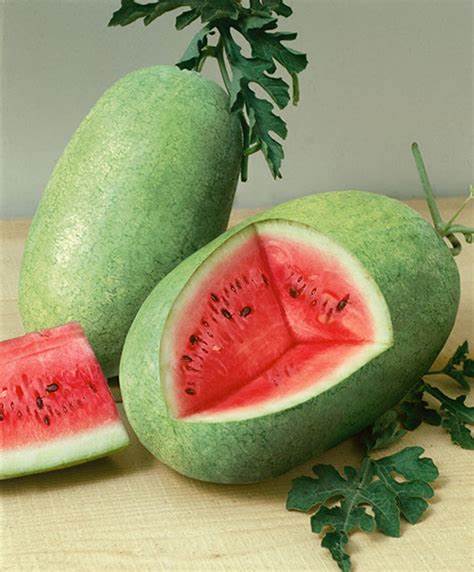 | Large oblong fruits with a light green exterior, pink-red flesh, and a thick and tough rind have resistance to FON race 0 | (Wu et al., 2019) |
| Shengnvhong 3  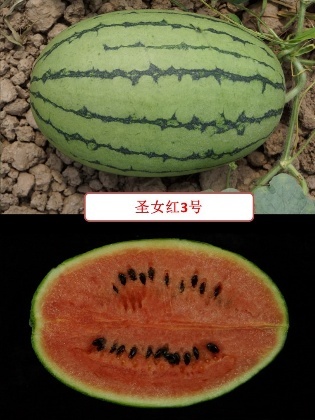 | A small-fruit variety and an early-maturing variety; oval fruit covered with thin stripes; pink flesh; medium resistance to Fusarium wilt. | (Song et al., 2017) |
| Shenxuan 958  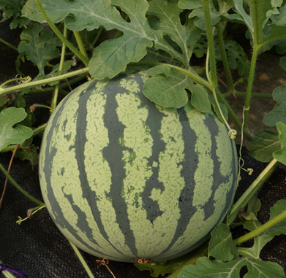 | Medium-sized and medium to early-maturing variety; round fruit; pink flesh; hard fruit skin, high sugar content in the flesh; high resistance to Fusarium wilt. | (Yang et al., 2018) |
| Shenkang 988  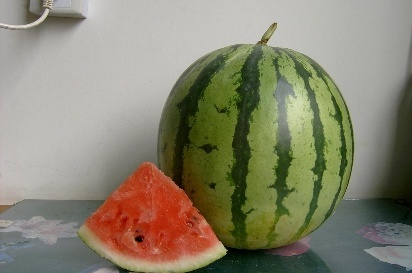 | An early-maturing large-sized variety; round shape and green striped skin with wide stripes; pink fruit, crisp and juicy; high resistance to Fusarium wilt. | (Gu et al., 2011) |
| Zaokangjingxin  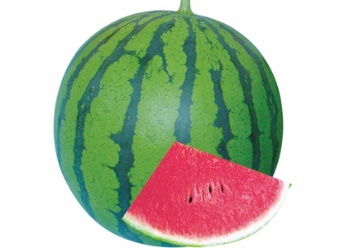 | An early-maturing medium-sized variety; strong fruit-setting ability; round shape and green striped skin with wide stripes; bright red flesh; juicy and good flavor; high resistance to Fusarium wilt. | (Yang et al., 2004) |
| Nongkeda 11  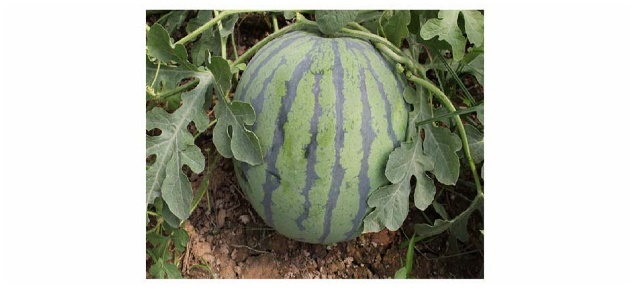 | Fruit is round with thin skin; flesh is red, crisp, and tastes good; high resistance to Fusarium wilt. | (Zhang et al., 2016) |
| Longsheng Jiali  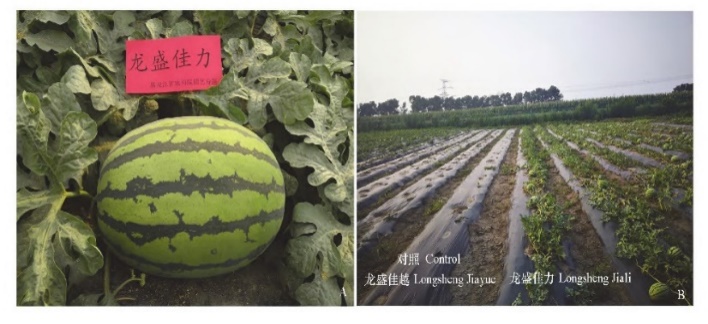 | The plant exhibits vigorous growth; easily sets fruit, elliptical fruits with waxy powder; individual fruit has a large weight; the flesh is fine, and the pulp is red. Classified as a mid-maturing variety with high resistance to FON race 1 and anthracnose. | (Wang et al., 2023) |
| Longke No.13  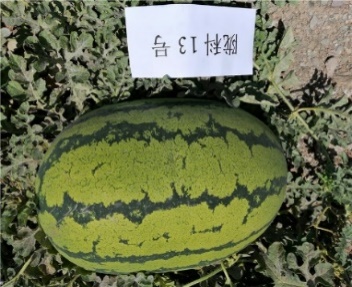 | Oval-shaped fruit with green and dark green skin; thin skin; tough in texture; resistant to storage and transportation; the flesh is red, fine, and crisp and tastes sweet. The variety sets fruit easily and is highly resistant to FON race 1. | (Su et al., 2024) |
| USVL-360  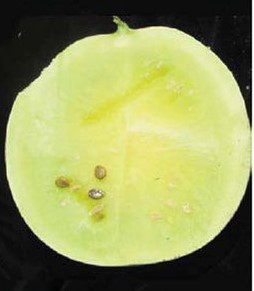 | Globular fruits with a light green-yellow rind and dense green flesh; large stem diameters; wide leaves with wide notched lobes and serrated edges; medium resistance to FON race 2. | (Levi et al., 2014) |
| USVL246-FR2  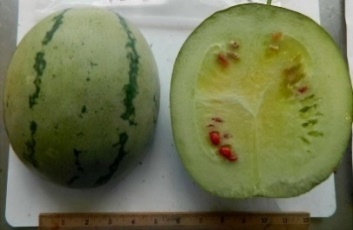 | Oblate fruits; light green fruit rind with dark green stripes; flesh is firm and salmon yellow with red/orange seeds; high resistance to FON race 2 | (Wechter et al., 2016) |
| USVL252-FR2  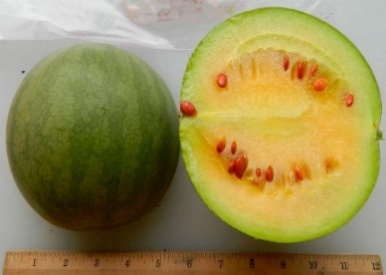 | round to slightly broad fruits; medium green fruit rind with light green stripes; the flesh is firm, and flesh is firm and white to salmon yellow with red/orange seeds; high resistance to FON race 2 | (Wechter et al., 2016) |

**References**

Gu, W., Song, R., Yang, H., Ma, K., 2011. A new watermelon F_1 hybrid—'Shenkang 988'. China Veget. (18), 102-104. http://dx.doi.org/10.19928/j.cnki.1000-6346.2011.18.023

Hao, W., Ren, L., Ran, W., Shen, Q., 2010. Allelopathic effects of root exudates from watermelon and rice plants on *Fusarium oxysporum* f.sp. *niveum*. Plant Soil 336(1-2), 485-497. http://dx.doi.org/10.1007/s11104-010-0505-0

Kou, J., Khashi U Rahman, M., Du, W., Yang, L., Li, D., Li, W., Pan, K., 2021. Effects of Exogenously applied palmitic acid on systemic resistance against *Fusarium oxysporum* f. sp. *niveum* in watermelon (*Citrullus lanatus* (Thunb.) Matsum. et Nakai). Allelopathy J. 52(2), 213-224. http://dx.doi.org/10.26651/allelo.j/2021-52-2-1317

Levi, A., Thies, J., Wechter, P., Farnham, M., Weng, Y., Hassell, R., 2014. USVL-360, a novel watermelon tetraploid germplasm line. Hortscience 49(3), 354-357. http://dx.doi.org/10.21273/HORTSCI.49.3.354

Li, H., Yuan, G., Zhu, C., Zhao, T., Zhang, R., Wang, X., Yang, J., Ma, J., Zhang, Y., Zhang, X., 2019. Soil fumigation with ammonium bicarbonate or metam sodium under high temperature alleviates continuous cropping-induced *Fusarium* wilt in watermelon. Sci. Hortic. 246, 979-986. http://dx.doi.org/10.1016/j.scienta.2018.11.090

Liu, B., Wu, F., Yang, Y., Wang, X., 2009. Amino acids in watermelon root exudates and their effect on growth of *Fusarium oxysporum* f.sp *nevium*. Allelopathy J. 23(1), 139-147.

Ma, K., Kou, J., Khashi U Rahman, M., Du, W., Liang, X., Wu, F., Li, W., Pan, K., 2021. Palmitic acid mediated change of rhizosphere and alleviation of Fusarium wilt disease in watermelon. Saudi J. Biol. Sci. 28(6), 3616-3623. http://dx.doi.org/10.1016/j.sjbs.2021.03.040

Ren, L., Huo, H., Zhang, F., Hao, W., Xiao, L., Dong, C., Xu, G., 2016. The components of rice and watermelon root exudates and their effects on pathogenic fungus and watermelon defense. Plant Signal. Behav. 11(6), e1187357. http://dx.doi.org/10.1080/15592324.2016.1187357

Song, R., Yang, H., Li, C., Zhu, L., Gu, W., 2017. Germplasm improvement and utilization for small-fruit watermelon with early maturity,good quality,and resistance to disease. Acta Agric. Shanghai 33(03), 96-100. http://dx.doi.org/10.15955/j.issn1000-3924.2017.03.18

Su, Y., Wang, Z., Ren, K., Gu, Y., 2024. A new watermelon F1 hybrid—‘Longke No.13’. China Veget. 1(10), 135-137+155. http://dx.doi.org/10.19928/j.cnki.1000-6346.2024.0055

Tong, Y., Zheng, X., Hu, Y., Wu, J., Liu, H., Deng, Y., Lv, W., Yao, H., Chen, J., Ge, T., 2024. Root exudate-mediated plant–microbiome interactions determine plant health during disease infection. Agric. Ecosyst. Environ. 370, 109056. http://dx.doi.org/10.1016/j.agee.2024.109056

Wang, X., Jia, Y., Yan, W., Fu, Y., You, H., Li, D., Zhao, J., 2023. A new watermelon cultivar 'Longsheng Jiali' with high resistance to Fusarium wilt. Acta Hortic. Sin. 50(2), 455-456. http://dx.doi.org/10.16420/j.issn.0513-353x.2022-0762

Wechter, W., McMillan, M., Farnham, M., Levi, A., 2016. Watermelon germplasm lines USVL246-FR2 and USVL252-FR2 tolerant to *Fusarium oxysporum* f. sp. *niveum* race 2. Hortscience 51(8), 1065-1067. http://dx.doi.org/10.21273/HORTSCI.51.8.1065

Wu, H., Raza, W., Fan, J., Sun, Y., Bao, W., Shen, Q., 2008a. Cinnamic acid inhibits growth but stimulates production of pathogenesis factors by *in Vitro* cultures of *Fusarium oxysporum* f.sp. *niveum*. J. Agric. Food Chem. 56(4), 1316-1321. http://dx.doi.org/10.1021/jf0726482

Wu, H., Liu, D., Ling, N., Bao, W., Ying, R., Shen, Q., 2009. Influence of root exudates of watermelon on *Fusarium oxysporum* f. sp. *niveum*. Soil Sci. Soc. Am. J. 73(4), 1150-1156. http://dx.doi.org/10.2136/sssaj2008.0266

Wu, H., Luo, J., Raza, W., Liu, Y., Gu, M., Chen, G., Hu, X., Wang, J., Mao, Z., Shen, Q., 2010. Effect of exogenously added ferulic acid on *in vitro* *Fusarium oxysporum* f. sp. *niveum*. Sci. Hortic. 124(4), 448-453. http://dx.doi.org/10.1016/j.scienta.2010.02.007

Wu, S., Wang, X., Reddy, U., Sun, H., Bao, K., Gao, L., Mao, L., Patel, T., Ortiz, C., Abburi, V., Nimmakayala, P., Branham, S., Wechter, P., Massey, L., Ling, K., Kousik, C., Hammar, S., Tadmor, Y., Portnoy, V., Gur, A., Katzir, N., Guner, N., Davis, A., Hernandez, A., Wright, C., McGregor, C., Jarret, R., Zhang, X., Xu, Y., Wehner, T., Grumet, R., Levi, A., Fei, Z., 2019. Genome of 'Charleston Gray', the principal American watermelon cultivar, and genetic characterization of 1,365 accessions in the US National Plant Germplasm System watermelon collection. Plant Biotechnol. J. 17(12), 2246-2258. http://dx.doi.org/10.1111/pbi.13136

Yang, X., Xu, J., Zhang, P., Gao, C., 2004. A new watermelon cultivar (combination) 'Zaokangjingxin' with high quality and high resistance to Fusarium wilt. Jiangsu J. Agric. Sci. 20(4), 253. http://dx.doi.org/10.3969/j.issn.1000-4440.2004.04.017

Yang, H., Zhu, L., LI, C., Song, R., 2018. A new disease-resistant and high quality watermelon cultivar 'Shenxuan 958'. Acta Agric. Shanghai 34(04), 74-78. http://dx.doi.org/10.15955/j.issn1000-3924.2018.04.15

Zhang, Y., Ma, J., Zhang, X., 2016. A new watermelon cultivar ‘Nongkeda 11’ with high resistance to Fusarium wilt. Acta Hortic. Sin. 43(1), 199-200. http://dx.doi.org/10.16420/j.issn.0513-353x.2014-1114
